# Long noncoding RNA LINC00899 suppresses breast cancer progression by inhibiting miR-425

**DOI:** 10.18632/aging.102426

**Published:** 2019-11-18

**Authors:** Wenying Zhou, Jiao Gong, Yaqiong Chen, Jiahao Chen, Qi Zhuang, Jing Cao, Zhixiong Mei, Bo Hu

**Affiliations:** 1 Department of Laboratory Medicine, Key Laboratory of Liver Disease of Guangdong Province, Third Affiliated Hospital of Sun Yat-sen University, Guangzhou, P.R. China; 2 Department of Infectious Diseases, Key Laboratory of Liver Disease of Guangdong Province, Third Affiliated Hospital of Sun Yat-sen University, Guangzhou, P.R. China; 3 Obstetrical Department, Key Laboratory of Liver Disease of Guangdong Province, Third Affiliated Hospital of Sun Yat-sen University, Guangzhou, P.R. China

**Keywords:** breast cancer, LINC00899, proliferation, miR-425, DICER

## Abstract

Long non-coding RNAs (lncRNAs) have emerged as important regulators in cancer, including breast cancer. The precise expression pattern of long noncoding RNA 00899 (LINC00899) in breast cancer and its mechanisms of action have not been reported. Here, we found that LINC00899 is downregulated in breast cancer tissues and cell lines. Kaplan-Meier analysis showed that elevated LINC00899 expression is closely associated with better relapse-free survival (RFS) in breast cancer, including the basal, luminal A or luminal B breast cancer subtypes. Gene Ontology terms and Kyoto Encyclopedia of Genes and Genomes pathway analysis suggested that LINC00899 is closely related to several cancer associated processes, including tight junction- and metabolism-associated pathways. Functional assays indicated that LINC00899 overexpression suppresses proliferation, migration and invasion of breast cancer cells in vitro. Moreover, LINC00899 was found to competitively bind miR-425, thereby functioning as a tumor suppressor by enhancing DICER1. Overexpression of miR-425 attenuated the LINC00899-induced inhibition of breast cancer cell proliferation and invasion. These findings highlight the important role of the LINC00899-miR-425-DICER1 axis in breast cancer cell proliferation and invasion, and could potentially lead to new lncRNA-based diagnostics or therapeutics for breast cancer.

## INTRODUCTION

Breast cancer is the most common cancer among women in China [1]. Its high mortality rate also makes breast cancer the leading cause of cancer deaths in China. In addition, there is reportedly an upward trend in the incidence and mortality rates for breast cancer [2, 3]. Mortality in breast cancer reflects the interruption of normal biological function due to tumor progression and metastasis [4, 5]. However, the molecular mechanisms underlying those processes remain unclear.

Long noncoding RNAs (lncRNAs) are a category of noncoding RNAs composed of more than 200 nucleotides [6–8]. Current evidence indicates that LncRNAs play important roles in various human cancers, including breast cancer [9], liver cancer [10] and gastric cancer [11], among others. LncRNAs have been shown to regulate cancer cell proliferation, migration, invasion [12]. For instance, SNHG16 promotes breast cancer progression by competitively binding miR-98, which targets transcription factor E2F5 [13]. LINC00899 is a newly identified lncRNA encoded on chromosome 22q13.31. Wang et al first reported that serum LINC00899 levels could be a potentially useful noninvasive biomarker for early clinical detection and prognosis of acute myeloid leukemia [14]. However, the potential effect and mechanism of LINC00899 on breast cancer remains unclear.

In the present study, we investigated role of LINC00899 in the progression of breast cancer and explored the underlying mechanisms. We found that LINC00899 is downregulated in human breast cancer tissues and cell lines, that its overexpression suppresses cell proliferation and invasion, and that its antitumor activity during breast cancer development reflects its ability to disrupt miR-425-mediated suppression of DICER1. These results may provide a new insight for the treatment of breast cancer.

## RESULTS

### LINC00899 is down-regulated in breast cancer tissues and cells

**Figure 1:**
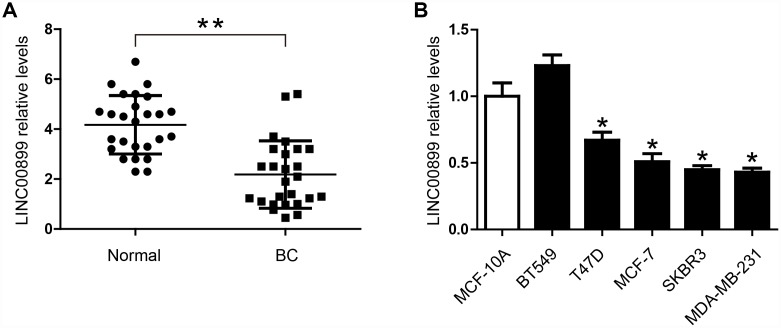
**LINC00899 is downregulated in human breast cancer tissues and cell lines.** (**A**) Comparison of the relative LINC00899 expression levels between 26 pairs of breast cancer (BC) tissues and adjacent normal tissues. (**B**) Comparison of the relative LINC00899 expression levels between the normal human mammary epithelial cells (MCF-10A) and 5 BC cell lines. Data presented as means ± SD, *p< 0.05, **p< 0.01.

To investigate the role of LINC00899 in breast cancer, we analyzed its expression in the cancer tissues and adjacent normal tissues using real-time qPCR. The results showed that LINC00899 expression was significantly decreased in breast cancer tissues (Figure 1A). In addition, qPCR was performed to determine LINC00899 levels in five human breast cell lines (BT549, T47D, MCF-7, SKBR3 and MDA-MB-231) and the MCF-10A normal breast cell line. As shown in Figure 1B, four of the breast cancer cell lines (not BT549 cells) exhibited apparently reduced levels of LINC00899, whereas MCF-10A cells showed high levels of LINC00899. These results suggest that LINC00899 is down-regulated in breast cancer cells.

### LINC00899 down-regulation is associated with shorter survival time

**Figure 2:**
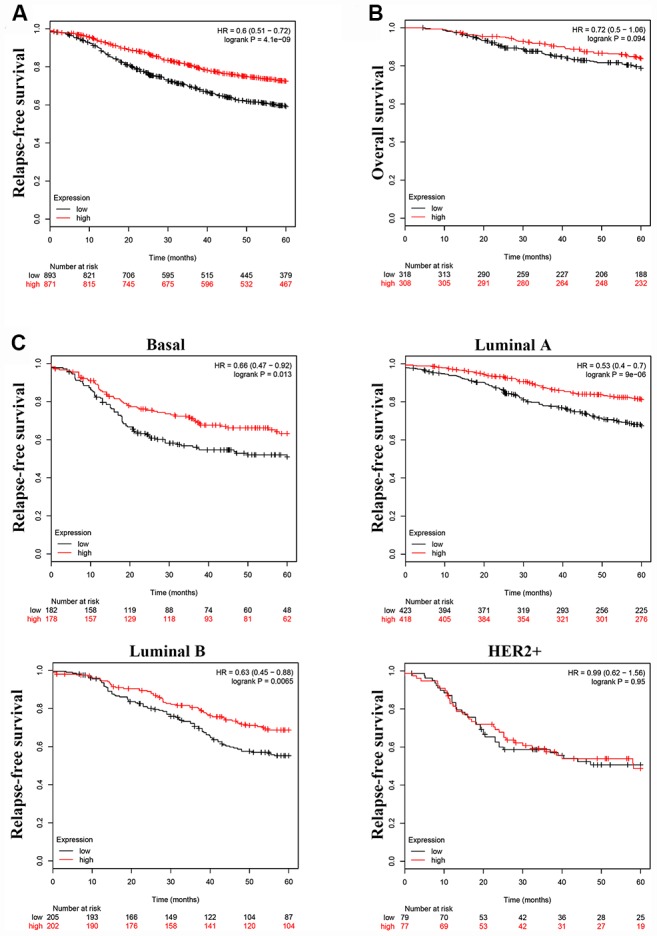
**High LINC00899 levels correlate with a better prognosis in breast cancer patients.** (**A**–**C**) Kaplan–Meier survival curve analysis performed using KM plotter. Breast cancer patients were divided into two equal groups based on median expression value of LINC00899. The hazard ratio (HR) and log-rank p-value comparing the two groups are shown. Low and high risks are indicated in black and red, respectively.

To investigate whether LINC00899 levels correlate with patient prognosis and relapse-free survival (RFS), we analyzed the prognostic value of LINC00899 in a large public clinical microarray database using the Kaplan–Meier plotter (http://kmplot.com/). The breast cancer patients were split into two equal groups based on median LINC00899 expression and compared using Kaplan–Meier survival analysis. Results showed that high LINC00899 expression was significantly associated with longer RFS but not with overall survival (OS) (Figure 2A and 2B). When we then subdivided patients based on their breast cancer type (Basal, Luminal A, Luminal B, HER2+), we found that patients with Basal, Luminal A, or Luminal B cancers who exhibiting high LINC00899 expression lived longer (Figure 2C). This suggests LINC00899 levels have important prognostic value for breast cancer patients.

### Overepression of LINC00899 restrains breast cancer cell proliferation, migration and invasion in vitro

**Figure 3:**
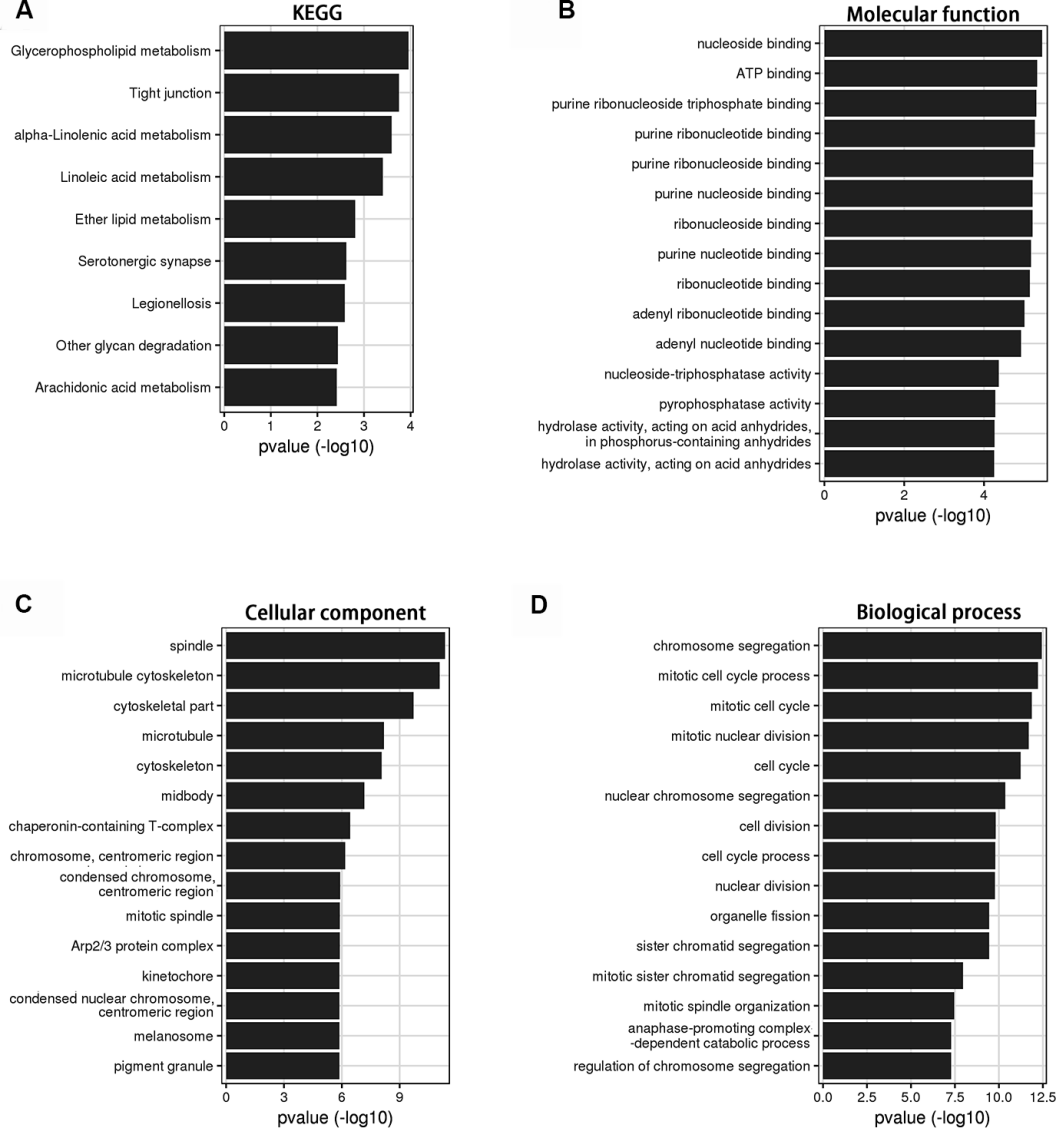
**KEGG and GO analysis of the genes relevant to LINC00899 in breast cancer.** (**A**) KEGG pathway enrichment for LINC00899. (**B**–**D**) Molecular functions, cell components and biological processes. These results were retrieved from the circlncRNAnet.

Bioinformatics analysis was used to investigate the role of LINC00899 in breast cancer. Using circlncRNAnet, we analyzed LINC00899-related signaling pathways. Gene ontology (GO) terms and Kyoto Encyclopedia of Genes and Genomes (KEGG) pathway analysis indicated that LINC00899 is closely related to several cancer-associated processes, including tight junction- and metabolism-associated pathways (Figure 3A–3D).

**Figure 4:**
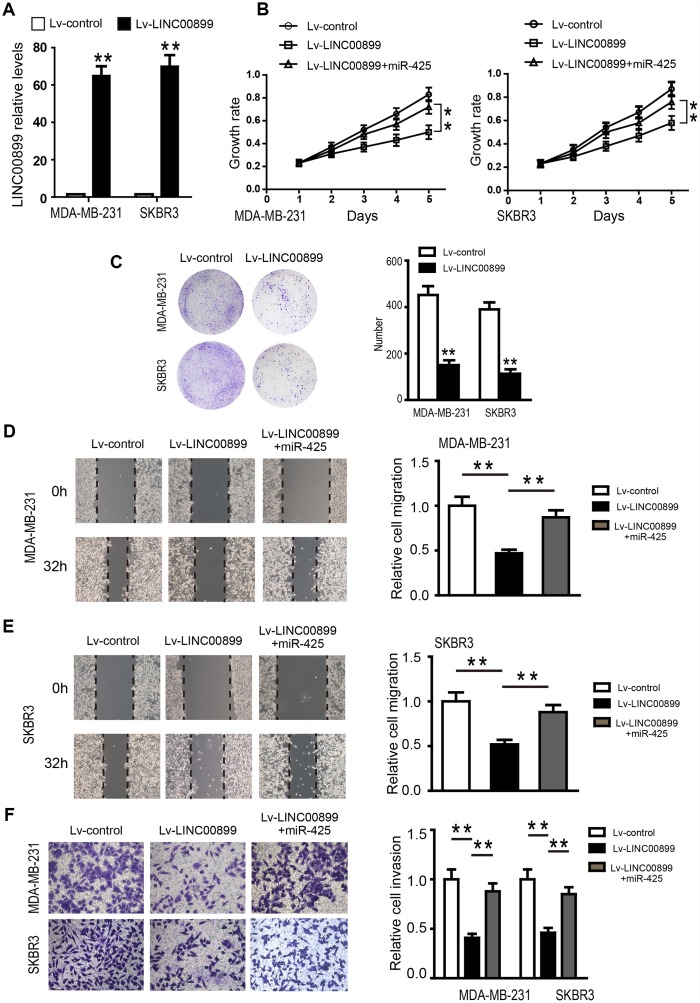
**Overexpression of LINC00899 inhibits BC cell proliferation, colony formation, migration and invasion.** (**A**) MDA-MB-231 and SKBR3 cells were transfected with LINC00899-carrying lentivirus (Lv-LINC00899) or control lentivirus (Lv-control) followed by qRT-PCR analysis of the relative LINC00899 expression levels. (**B**–**E**) Cells transfected with Lv-control, Lv-LINC00899 or Lv-LINC00899 plus miR-425 mimic. CCK8 (**B**) and colony formation (**C**) assays were used to assess proliferation of MDA-MB-231 and SKBR3 cells. Wound healing assays were performed to assess migration of MDA-MB-231 (**D**) and SKBR3 (**E**) cells. Transwell invasion assays were performed to assess invasion by MDA-MB-231and SKBR3cells (**F**). Data presented as means ± SD.**p< 0.01.

To investigate the specific function of LINC00899 in breast cancer, a LINC00899 expression vector or empty vector (control) were transfected into SKBR3 and MDA-MB-231 cells, which otherwise express LINC00899 only weakly. The significant increase of LINC00899 expression in these cells was confirmed by qRT-PCR (Figure 4A), and CCK-8 assays were used to detect SKBR3 and MDA-MB-231 cell proliferation. We found that proliferation of the two cell types was inhibited by LINC00899 overexpression as compared to their corresponding controls (Figure 4B). Similar results were observed with colony formation assays, which showed that LINC00899 overexpression reduced the ability of SKBR3 and MDA-MB-231 cells to form colonies in soft agar (Figure 4C). In wound healing assays, LINC00899 overexpression suppressed breast cancer cell migration. Similarly, Transwell assays with Matrigel showed that LINC00899 overexpression suppressed breast cancer cell migration and invasion (Figure 4D–4F). These data suggest that LINC00899 acts as a tumor suppressor in breast cancer.

### MIR-425 is a target of LINC00899 in breast cancer

**Figure 5:**
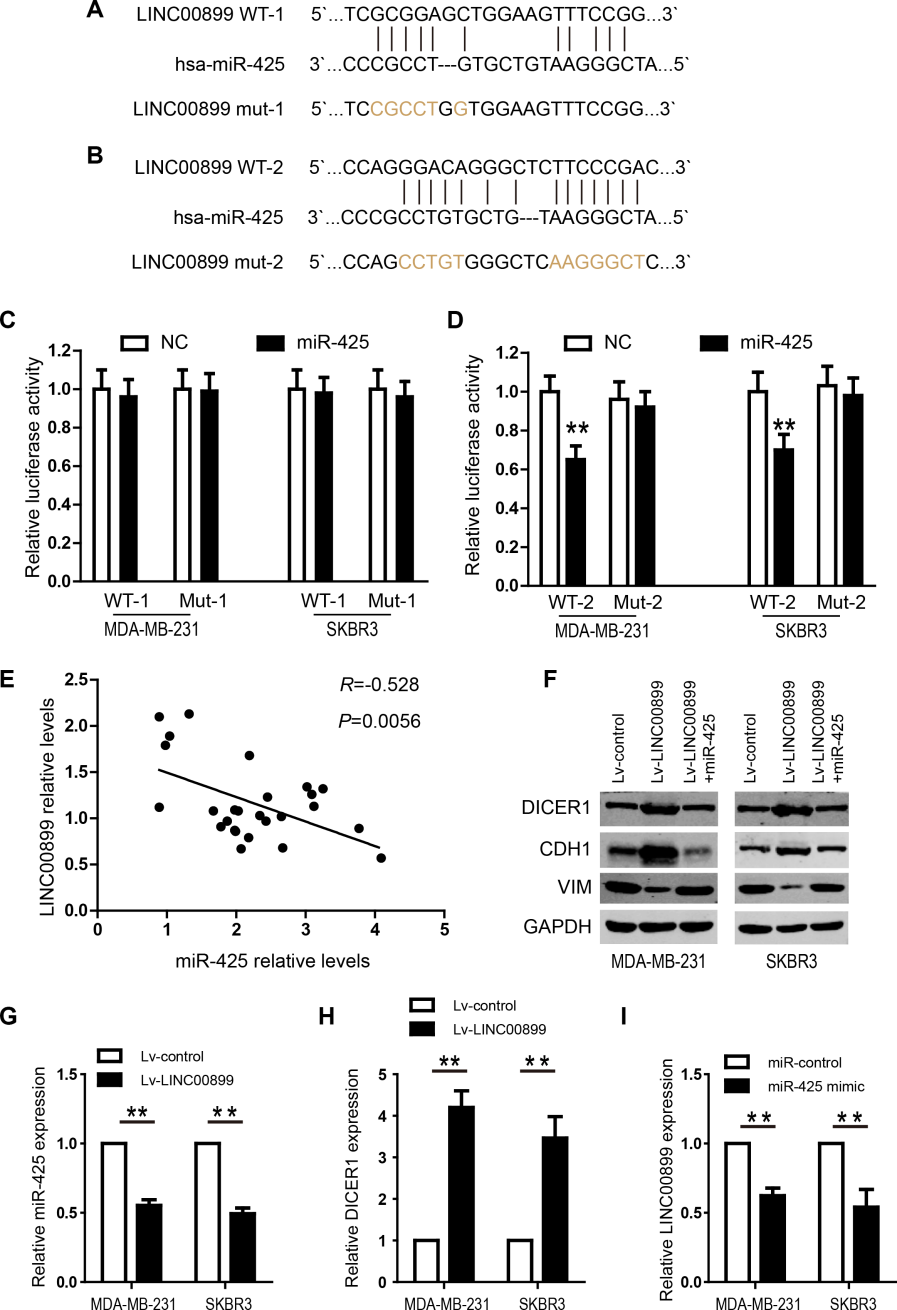
**LINC00899 directly binds to miR-425 in breast cancer.** (**A**–**B**) Bioinformatic analysis of the potential miR-425 binding sites within wild-type LINC00899-1 (LINC00899 WT-1) or LINC00899-2 (LINC00899 WT-2). (**C**–**D**) Luciferase reporter vectors containing WT-1 (left) or WT-2 (right) as well as corresponding mutant (Mut, Mut-1 or Mut-2) miR-425 binding sequences were cotransfected into BC cell lines (MDA-MB-231 and SKBR3) together with miR-425 mimic or NC mimic. Dual-luciferase reporter assays were performed to assess the luciferase activity in MDA-MB-231 and SKBR3 cells expressing luciferase fused to WT-LINC00899 or Mut-LINC00899. (**E**) Spearman’s analysis of the correlation between LINC00899 and miR-425 levels in BC tissues. Data are presented as means ± SD, *p< 0.01. (**F**) Western blot analysis of DICER1, CDH1 and VIM expression in MDA-MB-231 and SKBR3 cells transfected with Lv-control, Lv-LINC00899 or Lv-LINC00899 plus miR-425 mimic. (**G**–**H**) qRT-PCR analysis of miR-425 and DICER1 expression in MDA-MB-231 and SKBR3 cells transfected with Lv-control or Lv-LINC00899. (**I**) Expression of LINC00899 in cells transfected with miR-control or miR-425 mimic. Data presented as means ± SD, *p< 0.01.

To shed light on the potential mechanism by which LINC00899 exerts its regulatory functions in breast cancer, we predicted miRNAs that might interact with LINC00899 using the predication software miRcode and RNA22. Mir-425 was found to be a promising target of LINC00899, and the two predicted miR-425 binding sites in the LINC00899 sequence are showed in Figure 5A and 5B. Further validation revealed the second (Figure 5B) to be the true site mediating miR-425 binding to LINC00899. To verify the interaction between miR-425 and LINC00899, luciferase reporter vectors were constructed containing a wild-type (wt) or mutated (mut) miR-425 binding site in LINC00899. Dual-luciferase reporter assays then showed that miR-425 suppressed the luciferase activity of the LINC00899-wt reporter vector but barely affected the LINC00899-mut reporter vector (Figure 5C and 5D). Moreover, Spearman’s correlation analysis showed that LINC00899 levels correlated significantly with those of miR-425 in breast cancer tissue samples (Figure 5E). These data indicate that MIR-425 is a direct target of LINC00899 in breast cancer.

### LINC00899 inhibits breast cancer progression by inhibiting MIR-425-DICER1 interaction

We next investigated the role of miR-425 in LINC00899-driven inhibition of breast cancer progression by first transfecting miR-425 mimic into SKBR3 and MDA-MB-231 cells overexpressing LINC00899. The results showed that the LINC00899-mediated inhibition of breast cancer cell proliferation and invasion was reversed by transfection of miR-425 mimic (Figure 4B and 4D–4F). Clone formation experiments also showed that miR-425 restrains LINC00899-induced inhibitory effects on breast cancer cell growth (data not show). miR-425 reportedly promotes breast cancer proliferation and metastasis by targeting DICER1 [15]. With that in mind, we transfected SKBR3 and MDA-MB-231 cells with LINC00899 and co-transfected LINC00899 and miR-425 into another group of the cells, after which expression of DICER1, VIM and CDH1 was detected by Western blotting. The results showed that expression of DICER1 and CDH1 was enhanced while expression of VIM was decreased when LINC00899 was overexpressed (Figure 5F). However, those effects were reversed when LINC00899 and miR-425 were overexpressed together (Figure 5F). Moreover, qRT-PCR revealed that in breast cancer cells transfected with Lv-LINC00899 or Lv-control, the former downregulated miR-425 level but upregulated Dicer levels (Figure 5G–5H). Consistent with those results, overexpression of miR-425 mimic decreased LINC00899 expression (Figure 5I). These results suggest that LINC00899 acts as a tumor suppressor by inhibiting miR-425-mediated suppression of DICER1.

## DISCUSSION

Dysregulation of lncRNAs is reportedly involved in the tumorigenesis and progression of breast cancer, suggesting lncRNAs may be useful target for breast cancer diagnosis and therapy [16–18]. In the present study, we demonstrated that LINC00899 is downregulated in human breast cancer tissues and cell lines, and that overexpression of LINC00899 suppresses cell proliferation and invasion. Further mechanistic studies revealed that LINC00899 may act as a tumor suppressor that exerts its antitumor effects by disrupting miR-425-mediated suppression of DICER1 during breast cancer development. Moreover, bioinformation analysis showed that high LINC00899 expression was significantly correlated with good prognosis, which is indicative of the prognostic value of LINC00899 for breast cancer patients.

LINC00899, located on chromosome 22q13.31, is a newly identified lncRNA. LINC00899 knockdown in spinal ependymoma cells elevated levels of RBL2, p21, p27 and Bax; decreased levels of FoxO, Bcl-2, vimentin and annexin; reduced cell proliferation, migration and invasion; and enhanced apoptosis. Furthermore, FOXO inactivation due to loss of PTEN during melanoma development can mediate a senescence bypass. However, the details of the function and underlying mechanism of LINC00899 in breast cancer remain unclear. Here, qRT-PCR analysis revealed that LINC00899 is significantly down-regulated in breast cancer tissues and cell lines, while in vitro assays showed that overexpression of LINC00899 significantly inhibits breast cancer cell growth and metastasis. It is noteworthy that BT549 cells showed higher levels of LINC00899 than normal MCF-10A cells. We speculate that for breast cancer with BT549 as the main cancer cell type, LINC00899 may actually promote cancer progression.

MicroRNAs (miRNAs) are endogenous small RNAs (18–24 nucleotides) that are crucial regulators of mRNA expression. By binding of their 3′ untranslated regions (3′UTRs), miRNAs suppress translation of complimentary mRNAs [19]. Moreover, dysregulation of miRNAs reportedly leads to cancer tumorigenesis, progression and metastasis [20]. miR-425 is a highly conserved 23-nucleotide miRNA found on human chromosome 3. Previous studies have shown that by targeting PTEN miR-425 promotes cell proliferation and inhibits apoptosis in gastric cancer [21]. In breast cancer, miR-425 is overexpressed and promotes cell growth and invasion by suppressing DICER1.

It has been reported that lncRNAs often act as ceRNAs to regulate miRNAs by competitively binding common miRNAs. For instance, Wang et al. reported that DLEU1 contributes to ovarian carcinoma tumorigenesis and progression by interacting with miR-490-3p and altering CDK1 expression [22]. In addition, Cui et al. reported that the lncRNA CCAT1 promotes glioma tumorigenesis by sponging miR-181b, thereby suppressing its binding to endogenous targets FGFR3 and PDGFRα [23]. Here, we hypothesize that LINC00899 may also serve as a ceRNA to implement its biological function in breast cancer.

To investigate the correlation between LINC00899 and miRNA in breast cancer tumorigenesis, we performed a bioinformatics analysis and found that the miR-425 had a high probability of binding to LINC00899. We therefore hypothesized that LINC00899 might function as a miR-425 sponge to upregulate DICER1 expression. Consistent with that idea, luciferase reporter assays confirmed that LINC00899 directly combined to miR425 in breast cancer cells. LINC00899 significantly inhibited miR-425 expression, enhanced DICER1 and CDH1 expression, and reduced VIM expression. Furthermore, miR-425 mimic attenuated those inhibitory effects of LINC00899 and enhanced breast cancer cell proliferation, migration and invasion. These findings suggest that the miR-425-DICER1 interaction is crucial for malignant progression of breast cancer and that LINC0089 inhibits that interaction.

Taken together, our data reveal that LINC00899 serves as a tumor suppressor restraining breast cancer cell growth and metastasis. We found that LINC00899 sponges miR-425, thereby boosting expression of DICER1 during breast cancer progression. This suggests the LIN00899-miR-425-DICER1 axis may be a useful new therapeutic target in breast cancer. Additional studies are needed to assess the expression and function of LINC00899 in various subtypes of breast cancer to verify the role of LINC00899 in breast cancer.

## MATERIALS AND METHODS

### Tissue specimens and cell culture

Twenty-six samples of BC tissue with corresponding adjacent normal tissues were collected from BC patients during surgery. All patients provided informed consent according to procedures approved by the Shanghai Tenth People’s Hospital Institutional Review Board (The Certificate Number: SHSY-IEC-KY-4.0/17-23/01). All tissue specimens were rapidly frozen in liquid nitrogen and then stored at −80°C until extraction of RNA.

The BT549, T47D, MCF-7, SKBR3 and MDA-MB-231 human breast cancer cell lines were purchased from the Institute of Biochemistry and Cell Biology of the Chinese Academy of Sciences (Shanghai, China). The MCF-10A normal human breast cell line was purchased from the American Type Culture Collection (ATCC) (Manassas, VA, USA). Cells were cultured in Dulbecco’s modified Eagle’s medium (DMEM; GIBCO, Grand Island, NY) supplemented with 10% of fetal bovine serum (FBS), 100 U/ml penicillin and 100 mg/ml streptomycin (Invitrogen, Carlsbad, CA, USA). The cells were maintained in a humidified incubator at 37°C and under an atmosphere containing 5% CO_2_.

### Transfection and lentivirus transduction

Oligonucleotide transfection was performed using Lipofectamine 2000 reagent (Invitrogen, Carlsbad, CA, USA). cDNAs encoding LINC00899 and miR-425 were cloned into the pCDH-CMV-MCS-EF1-coGFP construct (System Biosciences, CA, USA) to generate the pCDH-CMV-miR-425 and pCDH-CMV-LINC00899 expression vector. The packaged lentivirus particles were named Lv-LINC00899. The empty lentiviral vector (Lv-control) was used as a control. Recombinant lentivirus plasmids were used to infect cells with 5 mg/mL Polybrene (Sigma, St. Louis, MO, USA).

### Real-time quantitative PCR (qPCR) analysis

Total RNA was extracted from the tissues and cell lines using TRIzol reagent (Invitrogen, Carlsbad, CA, USA) according to the manufacturer’s instructions. RNA was reverse-transcribed to cDNA using a PrimeScript RT Reagent Kit (TaKaRa, Dalian, China). SYBR Premix Ex Taq (TaKaRa) was used to detect expression of LINC00899 and miR-425. PCR was carried out at least in triplicate, and the results were analyzed on an ABI 7500 Fast Real-Time PCR System (Applied Biosystems, Foster City, CA). Relative levels of LINC00899 and miR-425 expression were quantified based on the expression levels of GAPDH and U6, respectively. The relative expression levels were calculated using the 2^−ΔΔCT^ method.

### Cell proliferation, cell migration and invasion assays

Cell proliferation was detected using a Cell Counting Kit-8 (CCK-8; Dojindo, Tokyo, Japan). Cells were seeded into 96-well plates to a density of 5×10^3^ cells/ well and incubated in 37°C under 5% CO_2_. CCK-8 assay solution (10 μl) was then added to each well at the indicated time. The absorbance at 450 nm was measured using an enzyme immunoassay analyzer (Thermo Fisher Scientific, Shanghai, China). For anchorage-independent soft agar colony formation assays, cells were seeded into six-well plates to a density of 5 ×10^3^ cells per well and maintained. Colonies were then counted until foci were evident. Transwell chambers (8 μm pore size, Corning, Cambridge, MA, USA) were used to perform cell migration and invasion assays. Transfected cells (2×10^5^ cells/mL) were resuspended in 200 μL of serum-free medium and seeded into the upper chamber. The cells were placed on the top side of the membrane without (migration assays) or with Matrigel (BD Biosciences) precoating (invasion assays). After incubation at 37°C for 48 h, cells that had migrated or invaded to the lower side of the membrane were fixed in 20% methanol and stained with 0.1% crystal violet for 15 min. The cells were counted in five randomly selected visual fields under an inverted phase-contrast microscope (Olympus).

### Luciferase reporter assays

miR-425 was found to be a directly regulated target of LINC00899 using miRcode bioinformatics tools (http://www.mircode.org/). The theoretical binding sequence for miR-425 within the LINC00899 gene and its mutant sequence were cloned into the psiCHECK-2 vector (Promega, Madison, WI, USA) to construct a dual luciferase reporter plasmid. The wild-type (Wt) 3′-UTR fragment of LINC0089 and its mutant (Mut) of the miR-425 binding site were cloned into a the psiCHECK-2 vector to form the reporter vectors Wt-LINC00899 and Mut-LINC00899, respectively. SKBR3 and MDA-MB-231 cells were transfected for 48 h with the Wt (or Mut) reporter plasmid and an NC mimic or miR-425 mimic. The luciferase activity was detected using a Dual Luciferase Reporter Gene Assay Kit (Beyotime Institute of Biotechnology, Shanghai, China) according to the manufacturer’s protocol. The relative luciferase activity was normalized to Renilla luciferase activity.

### Identifying LINC00899-related signaling pathways

LINC00899-related genes were predicted using online analysis tools, such as circlncRNAnet (http://app.cgu.edu.tw/circlnc/). The biological pathways were the identified via GO and KEGG pathway enrichment analyses using circlncRNAnet. A FDR <0.05 and P < 0.001 were set as the cutoff criteria.

### Statistical analysis

Statistical analyses were performed using SPSS 20.0 (SPSS Inc., Chicago, IL, USA). Data are presented as the mean ± standard deviation (SD) of at least three independent experiments. Differences between two groups were evaluated using Student’s t test. Differences among more than two groups were evaluated using one-way analysis of variance (ANOVA). Spearman rank-correlation was performed to calculate the correlation coefficient between LINC00899 and miR-425 expression levels.
